# Stakeholder Perspectives on Providing Person‐Centered Dementia Care in Low‐Resource Long‐Term Care Settings

**DOI:** 10.1002/alz.086624

**Published:** 2025-01-09

**Authors:** Sarah D Holmes, Alison Rataj, Nancy Kusmaul, Jing Wang, Yoon Chung Kim, Briana Murray, Laura Davie, Michael Lepore, Kirsten Corazzini

**Affiliations:** ^1^ University of Maryland Baltimore, Baltimore, MD USA; ^2^ University of New Hampshire, Durham, NH USA; ^3^ University of Maryland Baltimore County, Baltimore, MD USA; ^4^ 4 Library Way, Durham, NH USA; ^5^ University of Maryland School of Nursing, Baltimore, MD USA; ^6^ University of Massachusetts Amherst, Amherst, MA USA

## Abstract

**Background:**

There is a growing number of residents living with dementia (RLWD) in long‐term care (LTC) settings, but dementia care access and quality are more limited in communities with fewer health resources and these limitations are exacerbated by current dementia care workforce shortages. Low‐resource LTC settings, including poorer urban and rural settings, serve older adults who are at high risk for health inequities in dementia care. These settings can experience barriers in providing quality dementia care due to their limited ability to capture pertinent information about residents’ needs and preferences and ensuring that information is known by dementia care staff. Although there is evidence about the consequences of care inequities for RLWD in low‐resource LTC settings, less is known about stakeholders’ perspectives about key issues and opportunities for improving dementia care. This study describes stakeholder perspectives regarding the collection and sharing of information about RLWD for supporting quality dementia care in low‐resource LTC settings.

**Method:**

This was a community‐based, participatory research study in four low‐resource LTC settings (two rural, two urban) in the United States. All four settings were in medically underserved areas with two rural settings in New Hampshire and two urban settings in Maryland. In‐depth semi‐structured interviews were conducted with a purposive sample of participants (8 administrators/leaders, 20 care staff, 20 RLWD, and 20 care partners). Interviews were audio recorded, transcribed, and thematically analyzed in NVivo12.

**Result:**

Themes were identified in four core topic areas: 1) identifying information about RLWD to support quality care; 2) finding and accessing information by the care team; 3) sharing information with RLWD; and 4) describing quality measures most relevant for RLWD. Within each theme, subthemes were identified providing greater detail of the process for identifying, collection, and sharing information for supporting quality dementia care.

**Conclusion:**

This study revealed complexities in providing care for RLWD in diverse low‐resource communities and generates directions for addressing dementia care inequities. The findings shed light on practical strategies used by low‐resource LTC settings and motivate future research on measuring dementia care quality and advancing capacity among RLWD, their families, and care staff for inclusive, person‐centered dementia care.

